# Prognostic and clinicopathological role of soluble programmed cell death ligand-1 in patients with diffuse large B-cell lymphoma: a meta-analysis

**DOI:** 10.3389/fonc.2025.1506799

**Published:** 2025-01-31

**Authors:** Hongbin Lu, Lulu Luo, Jie Mi, Min Sun, Huaping Wang, Zheng Wang, Wenwen Ding

**Affiliations:** ^1^ Department of Hematology, Wuxi Branch of Ruijin Hospital Shanghai Jiao Tong University School of Medicine, Wuxi, Jiangsu, China; ^2^ Suzhou Jsuniwell Medical Laboratory, Suzhou, Jiangsu, China; ^3^ Department of Oncology, Wuxi Branch of Ruijin Hospital Shanghai Jiao Tong University School of Medicine, Wuxi, Jiangsu, China

**Keywords:** PD-L1, circulating, survival, biomarker, meta-analysis

## Abstract

**Background:**

The significance of soluble programmed death protein ligand-1 (PD-L1) in predicting the prognosis of diffuse large B-cell lymphoma (DLBCL) has been previously analyzed, but with conflicting results. This study investigated the effect of soluble PD-L1 (sPD-L1) expression on the prognosis of patients with DLBCL.

**Methods:**

We comprehensively searched the Web of Science, PubMed, Embase, and CNKI databases between their inception and August 14, 2024. The value of sPD-L1 in predicting the overall survival (OS) and progression-free survival (PFS) of patients with DLBCL was analyzed by computing the combined hazard ratios (HRs) and 95% confidence intervals (CIs). Associations between sPD-L1 and the clinicopathological factors of DLBCL were explored by combining odds ratios (ORs) and 95%CIs.

**Results:**

Seven articles involving 826 patients were included in this meta-analysis. Based on our pooled data, elevated sPD-L1 was closely related to poor OS (HR = 2.81, 95%CI = 1.99–3.95, p < 0.001) and inferior PFS (HR = 3.16, 95%CI = 1.41–7.08, p = 0.005) of DLBCL. Moreover, based on the pooled data, higher sPD-L1 was significantly related to the Eastern Cooperative Oncology Group Performance Status Scale (ECOG PS) ≥2 (OR=4.10, 95%CI=1.82-9.24, p=0.001), clinical stage III-IV (OR = 3.30, 95%CI = 1.48–7.39, p = 0.004), elevated lactate dehydrogenase (LDH) levels (OR = 2.14, 95%CI = 1.07–4.30, p = 0.032), and the International Prognostic Index (IPI) score 3–5 (OR = 3.83, 95%CI = 1.91–7.68, p < 0.001) in DLBCL.

**Conclusion:**

According to our findings, a higher sPD-L1 level was a significant predictor of poor OS and PFS in patients with DLBCL. Elevated sPD-L1 levels are closely related to factors representing disease aggressiveness in DLBCL.

## Introduction

Diffuse large B-cell lymphoma (DLBCL) has the highest morbidity among lymphoid neoplasms, accounting for 30% of annual non-Hodgkin lymphoma (NHL) cases (1). DLBCL usually occurs in the elderly, and the median age at diagnosis is seven decades (2). Approximately 60% of DLBCL cases can be treated with regimens such as rituximab, cyclophosphamide, doxorubicin, vincristine, and prednisone (R-CHOP) (3). Although patients developing non-germinal center B cell (non-GCB) subtypes of DLBCL exhibit markedly worse prognosis than those with the GCB subtype, the R-CHOP regimen is the preferred choice for new DLBCL cases (4). Despite the effectiveness of chemotherapy in patients with DLBCL, the survival rate is less than 40%, with a 5-year survival of just 20–30% (1). Furthermore, approximately 40% of DLBCL patients relapse or develop resistance to treatment (5). Consequently, identifying efficient biomarkers for DLBCL prognosis is imperative.

A growing body of evidence suggests that programmed death protein-1 (PD-1)- and programmed death protein ligand-1 (PD-L1)-targeting immune checkpoint inhibitors (ICIs) are potent therapeutic options for many cancers (6). By interacting with PD-1, PD-L1 suppresses T-cell growth and activity, leading to immunological resistance (7). According to recent studies, soluble PD-L1 (sPD-L1) serves as a significant prognostic marker for various cancers, such as gastric cancer (8), hepatocellular carcinoma (9), prostate cancer (10), melanoma (11), and non-small cell lung cancer (NSCLC) (12). The efficiency of sPD-L1 in predicting DLBCL prognosis has been previously analyzed; however, inconsistent findings have been reported (13–19). For example, in some studies, higher sPD-L1 levels were significantly associated with a poor prognosis of DLBCL (14–16, 19). However, according to other researchers, sPD-L1 is not markedly related to survival outcomes of DLBCL (17). Consequently, a literature review was conducted before the present meta-analysis to analyze sPD-L1’s precise effect on prognosis in DLBCL. Moreover, the correlations between sPD-L1 and DLBCL clinicopathological factors were examined in this meta-analysis.

## Materials and methods

### Study guideline

This study was conducted according to the preferred reporting items for systematic reviews and meta-analyses (PRISMA) guideline (20).

### Literature retrieval

We thoroughly searched PubMed, Web of Science, Embase, and CNKI between inception and August 14, 2024, using the search terms (soluble or serum or plasma) and (lymphoma large B-cell or diffuse large B-cell lymphoma or DLBCL or lymphoma) and programmed cell death ligand 1 (PD-L1). No limitations were placed on the language of publication. References from relevant studies were searched to identify additional related studies.

### Eligibility criteria

Articles on the following subjects were enrolled (1): those describing DLBCL diagnosed by pathology (2), those evaluating associations between sPD-L1 levels and survival outcomes in DLBCL (3), those with reported hazard ratios (HRs) and 95% confidence intervals (CIs), and (4) those with an available threshold sPD-L1. The following articles were excluded (1): reviews, case reports, comments, meeting abstracts, and letters (2); articles without available survival information; and (3) animal studies.

### Data acquisition and quality assessment

Two independent researchers (HL and LL) reviewed the literature and collected data from relevant studies. Disputes were resolved through negotiation with a third researcher (WD). The following information was collected: author, country, year, sample size, age, sex, study design, study center, study period, clinical stage, treatment, threshold, threshold determination method, specimen, follow-up, survival outcomes, survival analysis types, and HRs with 95%CIs. Primary and secondary survival endpoints included overall survival (OS) and progression-free survival (PFS), respectively. The Newcastle Ottawa Scale (NOS) was used to assess the quality of the selected articles (21). Notably, NOS evaluates quality in 3 domains, namely, selection, comparability, and outcome. The NOS scores range from 0–9, and scores ≥ 6 represent high-quality studies.

### Statistical analysis

The effect of sPD-L1 in forecasting OS and PFS in DLBCL was estimated by combining HRs and 95%CIs. We assessed the heterogeneity between the included studies based on I^2^ statistics and the Cochrane Q test. High heterogeneities were judged based on I^2^ > 50% and p < 0.10 and in those cases a random-effects model was used. Otherwise, a fixed-effects model was adopted. Subgroup analyses based on various factors were performed to further investigate the prognostic value of sPD-L1 expression. Associations between sPD-L1 and clinicopathological factors of DLBCL were analyzed by combining odds ratios (ORs) with 95% confidence intervals (CIs). To assess the stability and robustness of the results, we performed a sensitivity analysis by eliminating one study and calculating new HRs and 95%CIs. Publication bias was evaluated using funnel plots, and Begg’s and Egger’s tests. Stata 12.0 (StataCorp, College Station, Texas, USA) was used for statistical analysis. P-values < 0.05 were considered statistically significant.

## Results

### Study retrieval

**Figure 1:**
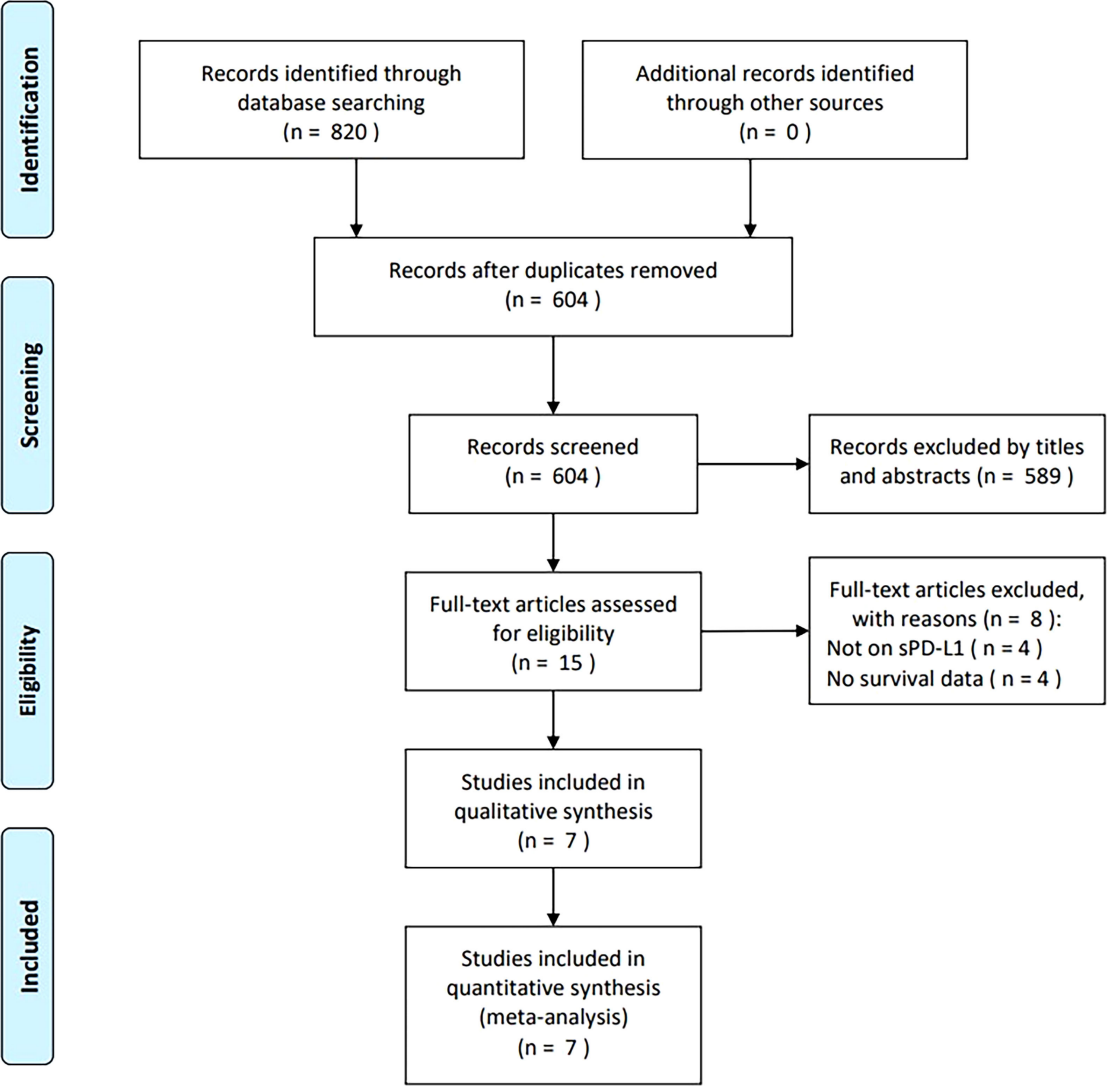
PRISMA flowchart of the study retrieval and selection process for this meta-analysis. From primary literature retrieval, 820 articles were obtained, among which, 604 were maintained following eliminating duplicates. By title- and abstract-screening, 589 articles were eliminated because of irrelevance. Later, full-texts in 15 articles were analyzed, among which, 8 were removed because of irrelevance to sPD-L1 (n=4) and no survival data provided (n=4). Eventually, seven articles were recruited in the present work.

From primary literature retrieval, 820 articles were obtained, of which 604 were retained after eliminating duplicates (**Figure 1**). Through title and abstract screening, 589 articles were eliminated because of irrelevance. Later, the full-texts of 15 articles were analyzed, of which eight were removed because of irrelevance to sPD-L1 (n = 4) and no provided survival data (n = 4). Ultimately, seven articles comprising 826 cases (13–19) were included in the present study (**Figure 1**).

### Recruited study characteristics

**Table 1** shows the baseline characteristics of all included studies (13–19) published between 2014–2023. Four studies were conducted in China (14, 16, 18, 19) and one each in France (13), South Korea (15), and Finland (17). Four articles were published in English (13, 15–17) and three were published in Chinese (14, 18, 19). Five studies had a retrospective design (14–16, 18, 19), and two had a prospective design (13, 17). Four were single-center studies (14, 16, 18, 19) and three were multicenter studies (13, 15, 17). The sample sizes ranged from 41–288 (median, 109). All studies included patients with stage I–IV DLBCL (13–19). Six studies indicated treatment of patients with chemotherapy (13, 14, 16–19) and one study indicated chemoradiotherapy (CRT) (15). Four studies reported sPD-L1 detection in plasma (13, 14, 16, 19), and three in sera (15, 17, 18). The median sPD-L1 threshold was 1.52 (range 0.432–4.57) ng/ml. The threshold was determined by using receiver operating characteristic (ROC) curves in five studies (13–15, 18, 19), and two studies used median values (16, 17). All seven studies reported the significance of sPD-L1 in predicting OS (13–19), whereas four studies reported a relationship between sPD-L1 and PFS (15–17, 19) in DLBCL. Five studies derived HRs and 95%CIs through univariate analysis (14–18), whereas two adopted multivariate analyses (13, 19). NOS scores ranged from 7–9, suggesting that the enrolled articles were of high quality (**Table 1**).

**Table 1:** Baseline characteristics of included studies in this meta-analysis.

Study	Year	Country	Sample size	Gender (M/F)	Age (years) Median(range)	Study design	Study center	Study period	Clinical stage	Treatment	Cut-off value (ng/ml)	Specimen	Cut-off determination	Follow-up (months) Median(range)	Survival outcomes	Survival analysis	NOS score
Rossille, D (13).	2014	France	288	169/119	<50y: 148 ≥50y: 140	Prospective	Multicenter	2005-2009	I-IV	Chemotherapy	1.52	Plasma	ROC curve	41.4	OS	Multivariate	9
Xu, Y (14).	2019	China	112	62/50	59.6 (17–79)	Retrospective	Single center	2013-2017	I-IV	Chemotherapy	4.57	Plasma	ROC curve	1-18	OS	Univariate	8
Cho, I (15).	2020	South Korea	68	27/41	55 (20–77)	Retrospective	Multicenter	2009-2017	I-IV	CRT	0.432	Sera	ROC curve	1-96	OS, PFS	Univariate	8
Fei, Y (16).	2020	China	87	41/46	56 (21–83)	Retrospective	Single center	NR	I-IV	Chemotherapy	1.21	Plasma	Median value	60 (2–106)	OS, PFS	Univariate	7
Vajavaara, H (17).	2021	Finland	121	75/46	56 (21–65)	Prospective	Multicenter	2011-2019	I-IV	Chemotherapy	0.766	Sera	Median value	61 (40–85)	OS, PFS	Univariate	9
Zhou, H (18).	2022	China	41	19/22	<60y: 11 ≥60y: 30	Retrospective	Single center	2017-2019	I-IV	Chemotherapy	3.58	Sera	ROC curve	1-25	OS	Univariate	7
Liu, D (19).	2023	China	109	60/49	58 (16–83)	Retrospective	Single center	2019-2021	I-IV	Chemotherapy	4.54	Plasma	ROC curve	15 (7–28)	OS, PFS	Multivariate	8

CRT, chemoradiotherapy; ROC, receiver operating characteristics; OS, overall survival; PFS, progression-free survival; NOS, Newcastle-Ottawa Scale.

### sPD-L1 and OS

**Figure 2:**
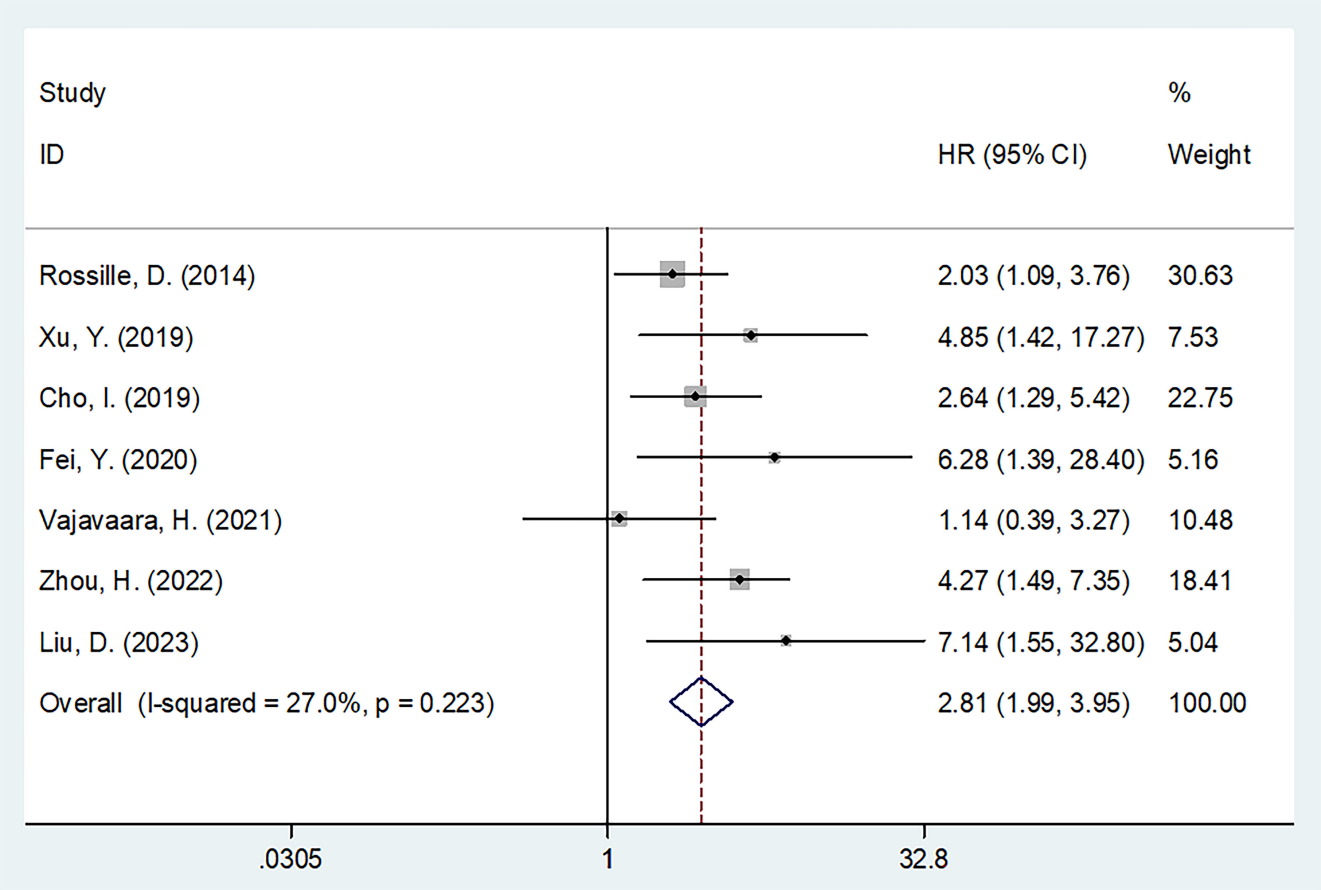
Forest plot of the association between sPD-L1 and OS in patients with DLBCL. HR=2.81, 95%CI=1.99-3.95, and p<0.001 could be acquired, which revealed that higher sPD-L1 level was markedly associated with dismal OS in DLBCL.

All seven studies with 826 patients (13–19) mentioned the effect of sPD-L1 in predicting the OS of DLBCL. A fixed-effects model was applied considering the insignificant heterogeneity (I^2^ = 27.0%, p = 0.223). Resulting values of HR = 2.81, 95%CI = 1.99–3.95, and p < 0.001 showed that a higher sPD-L1 level was markedly associated with poor OS in DLBCL (**Table 2**, **Figure 2**). Based on subgroup analyses, a higher sPD-L1 still significantly predicted poor OS regardless of region, sample size, study design, study center, treatment, cut-off value, or specimen (all p < 0.05; **Table 2**).

**Table 2:** Subgroup analysis of prognostic value of sPD-L1 for OS in patients with DLBCL.

Subgroups	No. of studies	No. of patients	Effects model	HR (95%CI)	p	Heterogeneity I^2^(%)	Ph
Total	7	826	Fixed	2.81(1.99-3.95)	<0.001	27.0	0.223
Geographic region							
Asia	5	417	Fixed	3.90(2.49-6.09)	<0.001	0	0.685
Non-Asia	2	409	Fixed	1.75(1.03-2.99)	0.040	0	0.354
Sample size							
<100	3	196	Fixed	3.52(2.13-5.83)	<0.001	0	0.496
≥100	4	630	Fixed	2.31(1.44-3.68)	<0.001	43.9	0.148
Study design							
Prospective	2	409	Fixed	1.75(1.03-2.99)	0.040	0	0.354
Retrospective	5	417	Fixed	3.90(2.49-6.09)	<0.001	0	0.685
Study center							
Single center	4	349	Fixed	4.98(2.82-8.80)	<0.001	0	0.930
Multicenter	3	477	Fixed	2.03(1.32-3.11)	0.001	0	0.433
Treatment							
Chemotherapy	6	758	Fixed	2.85(1.93-4.22)	<0.001	38.9	0.147
CRT	1	68	–	2.64(1.29-5.42)	0.008	–	–
Cut-off value (ng/ml)							
<1.5	3	276	Fixed	2.36(1.36-4.10)	0.002	43.6	0.170
≥1.5	4	550	Fixed	3.13(2.02-4.84)	<0.001	26.0	0.256
Specimen							
Serum	3	230	Fixed	2.64(1.64-4.26)	<0.001	47.8	0.147
Plasma	4	596	Fixed	2.99(1.83-4.89)	<0.001	29.6	0.235
Cut-off determination							
ROC curve	5	618	Fixed	2.99(2.06-4.34)	<0.001	4.9	0.379
Median value	2	208	Random	2.45(0.46-12.97)	0.293	69.8	0.069
Survival analysis							
Univariate	5	429	Fixed	3.04(1.98-4.66)	<0.001	28.4	0.232
Multivariate	2	397	Random	3.11(0.97-10.01)	0.057	55.4	0.134

CRT, chemoradiotherapy; ROC, receiver operating characteristics; OS, overall survival; PD-L1, programmed death-ligand 1; DLBCL, diffuse large B cell lymphoma.

### sPD-L1 and PFS

**Figure 3:**
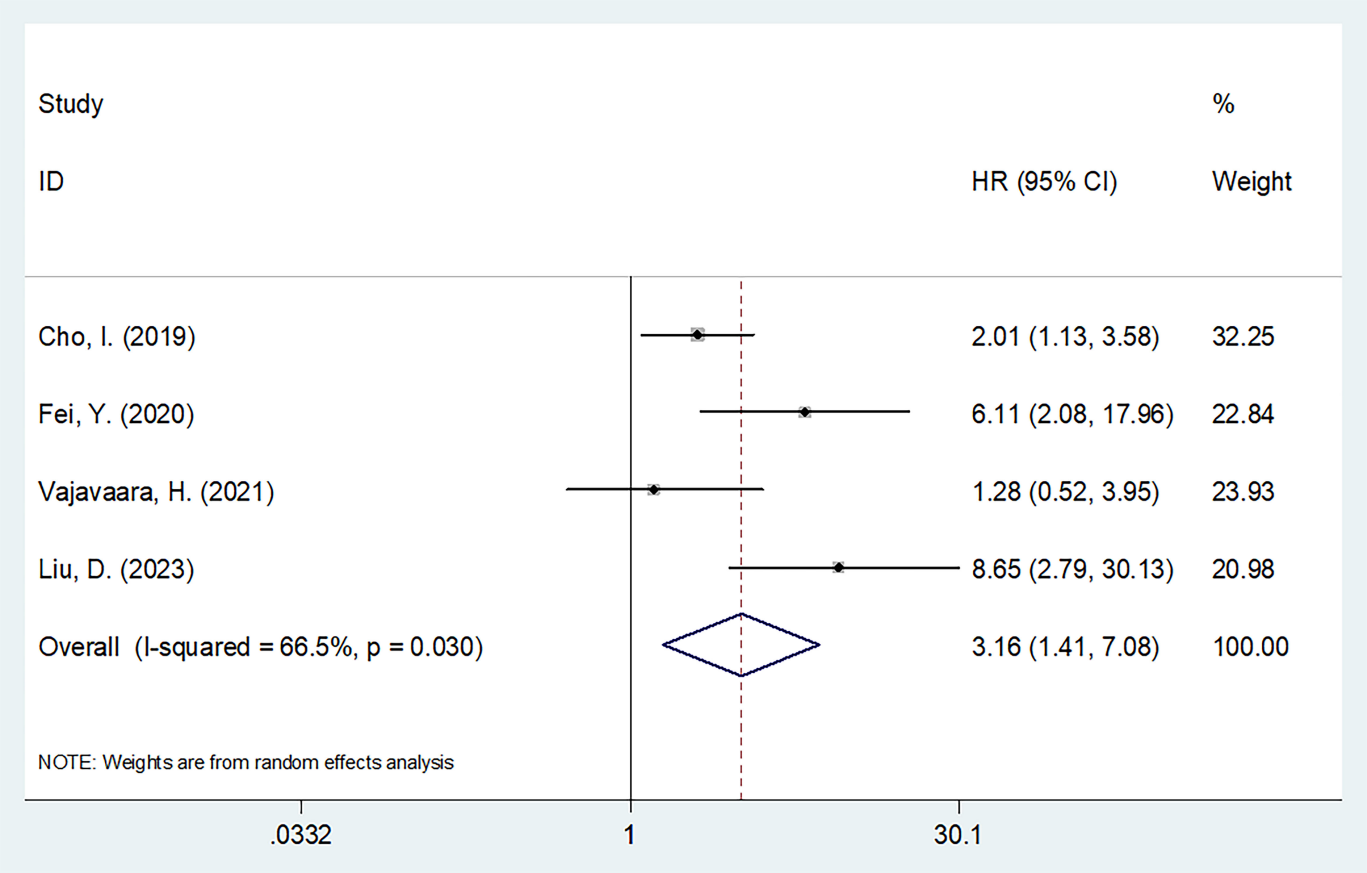
Forest plot of the association between sPD-L1 and PFS in patients with DLBCL. As a result, high sPD-L1 apparently estimated poor PFS of DLBCL (HR=3.16, 95%CI=1.41-7.08, p=0.005).

Four studies involving 385 patients reported a relationship between sPD-L1 levels and PFS (15–17, 19). Considering the heterogeneity (I^2^ = 66.5%, p = 0.035), this study utilized a random-effects model (**Table 2**). As a result, high sPD-L1 apparently estimated poor PFS of DLBCL (HR = 3.16, 95%CI = 1.41–7.08, p = 0.005; **Table 3**, **Figure 3**). As indicated by the subgroup analyses, the role of sPD-L1 in forecasting PFS remained unaffected by treatment, threshold, study center, specimen, or survival analysis (all p < 0.05; **Table 3**).

**Table 3:** Subgroup analysis of prognostic value of sPD-L1 for PFS in patients with DLBCL.

Subgroups	No. of studies	No. of patients	Effects model	HR (95%CI)	p	Heterogeneity I2(%)	Ph
Total	4	385	Random	3.16 (1.41-7.08)	0.005	66.5	0.035
Geographic region							
Asia	3	264	Random	4.25 (1.61-11.24)	0.004	69.4	0.038
Non-Asia	1	121	–	1.28 (0.48-3.53)	0.635	–	–
Sample size							
<100	2	155	Random	3.18 (1.09-9.28)	0.034	68.5	0.075
≥100	2	230	Random	3.24 (0.50-21.08)	0.218	82.6	0.017
Study design							
Prospective	1	121	–	1.28 (0.46-3.53)	0.635	–	–
Retrospective	3	264	Random	4.25 (1.61-11.24)	0.004	69.4	0.038
Study center							
Single center	2	196	Fixed	7.15 (3.21-15.89)	<0.001	0	0.671
Multicenter	2	189	Fixed	1.80 (1.09-2.98)	0.021	0	0.446
Treatment							
Chemotherapy	3	317	Random	3.96 (1.22-12.89)	0.022	71.4	0.030
CRT	1	68	–	2.01 (1.13-3.58)	0.017	–	–
Cut-off value (ng/ml)							
<1.5	3	276	Random	2.39 (1.11-5.13)	0.026	56.8	0.099
≥1.5	1	109	–	8.65 (2.63-28.43)	<0.001	–	–
Specimen							
Serum	2	189	Fixed	1.80 (1.09-2.98)	0.021	0	0.446
Plasma	2	196	Fixed	7.15 (3.21-15.89)	<0.001	0	0.671
Cut-off determination							
ROC curve	2	177	Random	3.79 (0.92-15.60)	0.065	78.6	0.031
Median value	2	208	Random	2.77 (0.60-12.80)	0.193	76.6	0.039
Survival analysis							
Univariate	3	276	Random	2.39 (1.11-5.13)	0.026	56.8	0.099
Multivariate	1	109	–	8.65 (2.63-28.43)	<0.001	–	–

CRT, chemoradiotherapy; ROC, receiver operating characteristics; PFS, progression-free survival; PD-L1, programmed death-ligand 1; DLBCL, diffuse large B cell lymphoma.

### Associations of sPD-L1 with clinicopathological features

**Figure 4:**
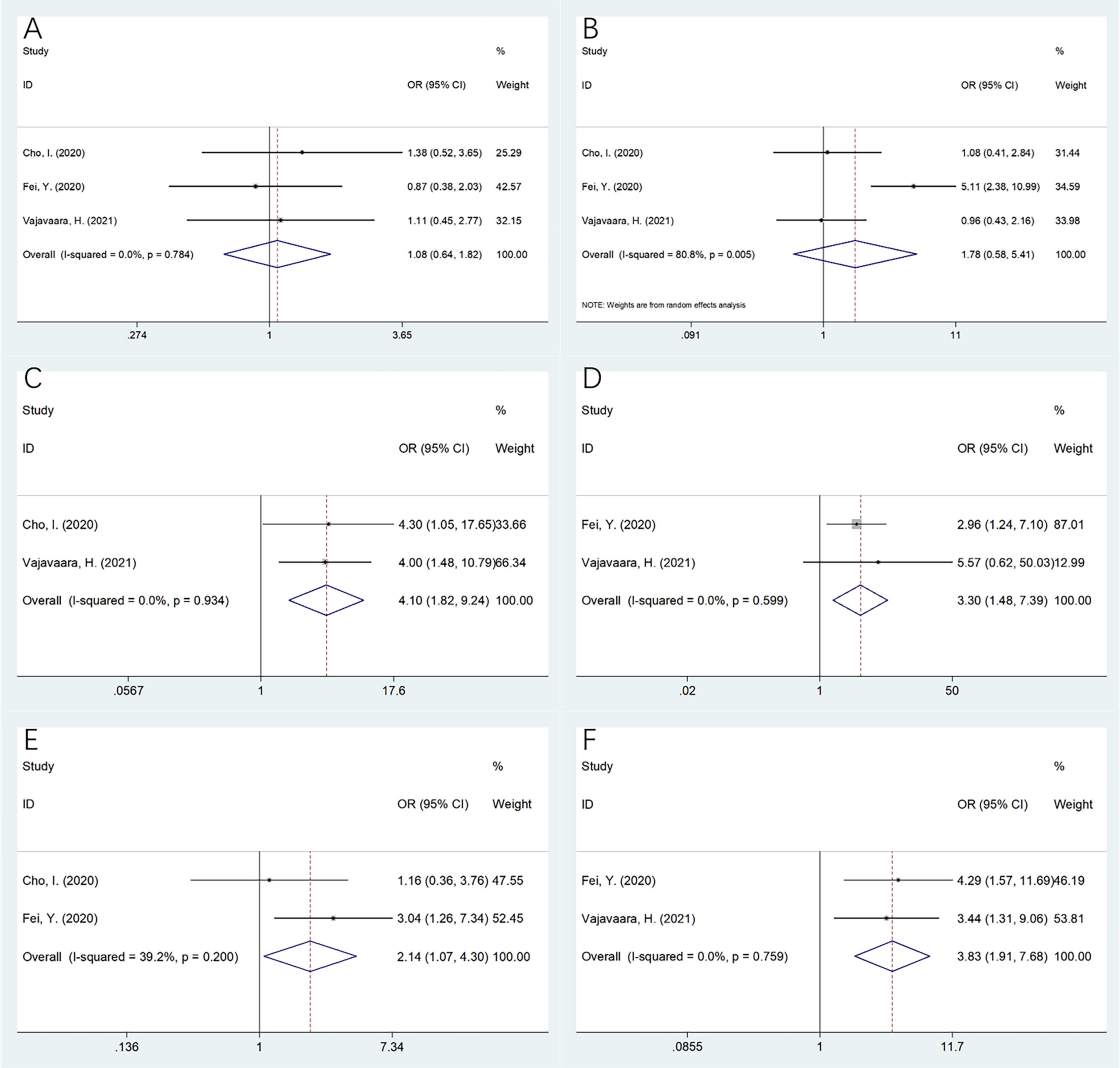
The relationship betweensPD-L1 and clinicopathological features in DLBCL. **(A)** Gender (male vs female); **(B)** Age (years) (≥60 vs <60); **(C)** ECOG PS (≥2 vs 0-1); **(D)** Clinical stage (III-IV vs I-II); **(E)** LDH level (elevated vs normal); and **(F)** IPI score (3-5 vs 0-2). As revealed by our pooling data, higher sPD-L1 was significantly related to ECOG PS≥2 (OR=4.10, 95%CI=1.82-9.24, p=0.001), clinical stage III-IV (OR=3.30, 95%CI=1.48-7.39, p=0.004), elevated LDH levels (OR=2.14, 95%CI=1.07-4.30, p=0.032), and IPI score 3-5 (OR=3.83, 95%CI=1.91-7.68, p<0.001). But sPD-L1 remained uncorrelated with gender (OR=1.08, 95%CI=0.64-1.82, p=0.778) or age (OR=1.78, 95%CI=0.58-5.41, p=0.312) in DLBCL.

Three studies incorporating 276 patients (15–17) provided information regarding the relationship between sPD-L1 expression and the clinicopathological features of DLBCL. As revealed by our pooled data, a higher sPD-L1 was significantly related to the Eastern Cooperative Oncology Group Performance Status (ECOG PS) ≥2 (OR = 4.10, 95%CI= 1.82–9.24, p = 0.001), clinical stage III–IV (OR= 3.30, 95%CI = 1.48–7.39, p = 0.004), elevated lactate dehydrogenase (LDH) levels (OR = 2.14, 95%CI = 1.07–4.30, p = 0.032), and the International Prognostic Index (IPI) score 3–5 (OR = 3.83, 95%CI= 1.91–7.68, p < 0.001) (**Table 4**, **Figure 4**). But sPD-L1 remained uncorrelated with sex (OR = 1.08, 95%CI = 0.64–1.82, p = 0.778) or age (OR= 1.78, 95%CI = 0.58–5.41, p = 0.312) in DLBCL (**Table 4**, **Figure 4**).

**Table 4:** The association between sPD-L1 and clinicopathological features of patients with DLBCL.

Variables	No. of studies	No. of patients	Effects model	OR (95%CI)	p	Heterogeneity I^2^(%)	Ph
Gender (male vs female)	3	276	Fixed	1.08(0.64-1.82)	0.778	0	0.784
Age (years) (≥60 vs <60)	3	276	Random	1.78(0.58-5.41)	0.312	80.8	0.005
ECOG PS (≥2 vs 0-1)	2	189	Fixed	4.10(1.82-9.24)	0.001	0	0.934
Clinical stage (III-IV vs I-II)	2	208	Fixed	3.30(1.48-7.39)	0.004	0	0.599
LDH level (elevated vs normal)	2	155	Fixed	2.14(1.07-4.30)	0.032	39.2	0.200
IPI score (3-5 vs 0-2)	2	208	Fixed	3.83(1.91-7.68)	<0.001	0	0.759

DLBCL, diffuse large B cell lymphoma; ECOG PS, Eastern Cooperative Oncology Group performance status; LDH, lactate dehydrogenase; IPI, International Prognostic Index.

### Sensitivity analysis

**Figure 5:**
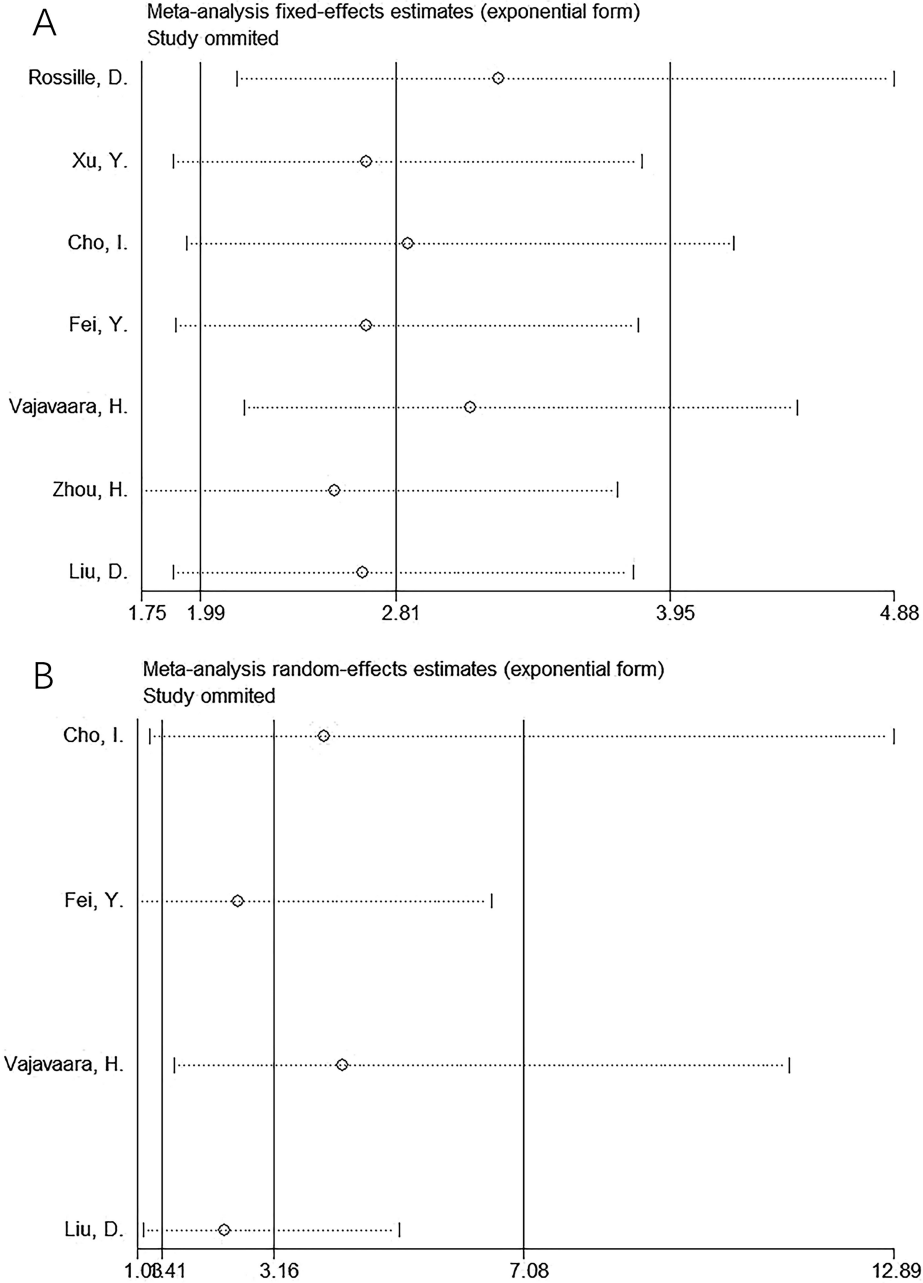
Sensitivity analysis. **(A)** OS and **(B)** PFS. One article was eliminated each time during sensitivity analyses on OS and PFS for analyzing the effect of this study on whole results. As a result, the results intuitively showed their robustness.

One article was eliminated during the sensitivity analyses of OS and PFS each time to analyze the effect of this study on the overall results. These results intuitively demonstrated their robustness (**Figure 5**).

### Publication bias

**Figure 6:**
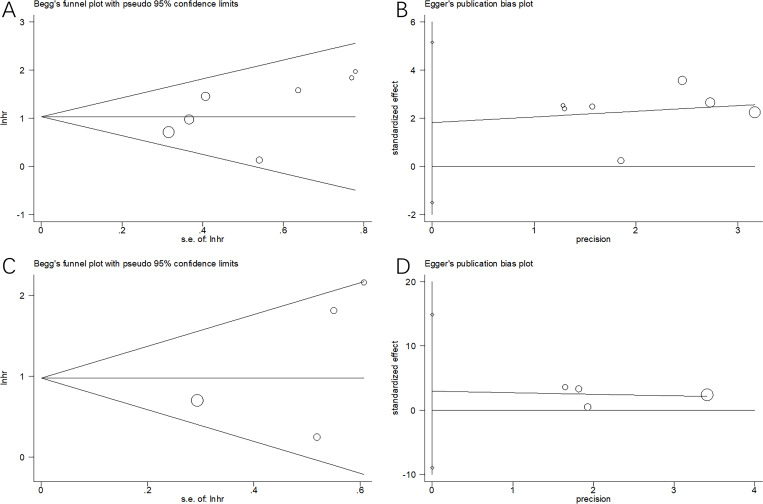
Publication bias test in this meta-analysis. **(A)** Begg’s test for OS, p=0.133; **(B)** Egger’s test for OS, p=0.219; **(C)** Begg’s test for PFS, p=0.308; and **(D)** Egger’s test for PFS, p=0.399. We conducted Begg’s and Egger’s tests to evaluate publication bias, which revealed no publication bias of OS or PFS.

Begg’s and Egger’s tests were conducted to evaluate publication bias, which revealed no publication bias for OS (p = 0.133 and 0.219 in Begg’s and Egger’s tests, respectively) or PFS (p = 0.308 and 0.399 in Begg’s and Egger’s tests, respectively) (**Figure 6**).

## Discussion

The effect of sPD-L1 in DLBCL prognosis has been widely examined; however, inconsistent results have been reported. Data were collected from seven articles with 826 cases (13–19) to analyze how sPD-L1 affects DLBCL prognosis. Based on our data, elevated sPD-L1 levels were markedly associated with unfavorable OS and shortened PFS in patients with DLBCL. Additionally, a higher sPD-L1 was also apparently connected to ECOG PS ≥ 2, advanced clinical stage, elevated LDH levels, and IPI score 3–5 in DLBCL. Sensitivity, subgroup, and publication-bias tests were used to verify the stability of the results. Collectively, high sPD-L1 levels remarkably predicted inferior short- and long-term survival in patients with DLBCL. To our knowledge, this is the first meta-analysis to explore the effect of sPD-L1 expression on DLBCL prognosis.

This study showed that sPD-L1 has a prognostic significance in DLBCL. The potential mechanisms underlying sPD-L1’s prognostic value for DLBCL are as follows: First, sPD-L1 can reduce cyclin A, ERK (p-ERK), and Akt; reduce adenosine triphosphate production; and attenuate T-cell respiration (22). By inhibiting the antitumor action of T cells by binding to PD-1 on their surface, sPD-L1 induces T cell death, thus promoting the immune escape of cancer cells (23). Second, CD274 encodes sPD-L1, a type I transmembrane glycoprotein. Its transcription generates several PD-L1 splice variants, each differing in length. In particular, exon 4-enriched variants produce PD-L1, which is secreted by the body (24). Third, in a mouse model of triple-negative breast cancer, a senescent tumor-cell vaccine expressing sPD-1 retarded tumor occurrence and inhibited tumor development (25).

Our meta-analysis showed that sPD-L1 is an important factor in predicting poor OS and PFS outcomes in patients with DLBCL. Therefore, the PD-1/PD-L1 axis may be an effective target for DLBCL treatment. Two antibodies targeting PD-1, nivolumab, and pembrolizumab, and three antibodies targeting PD-L1, durvalumab, atezolizumab, and avelumab, have been approved for B-cell lymphoma (26, 27). Atezolizumab is a monoclonal antibody of the humanized immunoglobulin G1 type that targets PD-L1, and has shown antitumor activity in several types of tumors. Younes et al. assessed the safety and effectiveness of atezolizumab combined with R-CHOP in patients with newly diagnosed DLBCL and found that the complete remission rates were enhanced when compared to those in the control group (28). PFS was achieved with a relatively shorter follow-up period than OS. Our meta-analysis showed consistent prognostic efficiency of sPD-L1 in both OS and PFS, indicating that sPD-L1 could be used to monitor long-term survival outcomes in DLBCL.

This meta-analysis suggests that sPD-L1 is a significant prognostic marker for DLBCL. This study had several strengths. To our knowledge, this is the first meta-analysis to investigate the prognostic value of sPD-L1 in DLBCL. Previous studies showed the prognostic effect of sPD-L1 in lymphoma (29); however, lymphoma is a heterogeneous disease with various entities. This meta-analysis focused on DLBCL, which accounts for 30% of all NHL cases. Second, this study not only investigated the prognostic value of sPD-L1 in DLBCL but also showed a correlation between sPD-L1 expression and several clinicopathological factors of DLBCL. These results provide a comprehensive understanding of the biological role of sPD-L1 in DLBCL. Third, our results were verified using sensitivity analysis and publication bias tests, and were reliable.

Recent studies have revealed advances in the prognostic function of sPD-L1 in cancer (30). A meta-analysis of 1,054 patients showed that elevated sPD-L1 levels were significantly associated with poor OS in patients with ICI-treated cancer (31). Mazzaschi et al. conducted a study on 109 patients with NSCLC, analyzing the pretreatment levels of soluble PD-L1, circulating PD1+ CD8+ cells, and NK cells as biomarkers for predicting ICI response (32). With the advent of precision medicine, liquid biopsies and circulating biomarkers have become essential for identifying the best treatment options for patients. Circulating biomarkers show potential for forecasting immunotherapy outcomes; however, they face hurdles before being integrated into standard clinical practice. First, no standardized pipelines are available for the isolation and analysis of circulating analytes. Second, no optimal cut-offs have been universally established for stratifying patients and predicting their response to ICI. Third, circulating biomarkers are more relevant for patients with advanced or metastatic disease, as those patients are more likely to release tumor-derived cells, EVs, and DNA fragments into the blood. The analysis of circulating biomarkers may not be beneficial in patients with localized tumors (33). Future studies should investigate the standard cut-off value of sPD-L1 in various cancers.

Recent meta-analyses have suggested that sPD-L1 is important in forecasting the prognostic outcomes of different cancers (34–38). According to Cui et al., higher sPD-L1 expression before treatment was markedly associated with poor OS and PFS in NSCLC patients in a meta-analysis of 928 patients (35). As reported by Scirocchi et al., higher sPD-L1 expression in the blood correlated with poor OS and PFS in tumor patients receiving immunotherapy in a meta-analysis of 12 articles (36). A recent meta-analysis of 1,188 cases showed that higher sPD-L1 levels were markedly associated with worse OS and PFS in patients with lung cancer receiving ICIs therapy (37). According to Li et al., higher sPD-L1 expression is associated with worse OS and other survival endpoints in different cancers in a meta-analysis of 21 studies (38). Our results are consistent with studies investigating other cancers.

The present study had some limitations. First, most eligible studies were conducted in Asia. Therefore, our findings are likely to be applicable to Asian patients with DLBCL. Second, most of the included studies had retrospective designs, and therefore, inherent heterogeneity was possible. Third, the threshold sPD-L1 level was not consistent among the eligible studies, which may have caused selection bias. Owing to these limitations, multicenter prospective trials with uniform thresholds should be conducted to validate the prognostic role of sPD-L1 in DLBCL.

## Conclusions

In summary, high sPD-L1 levels are a significant predictor of poor OS and PFS in patients with DLBCL. Elevated sPD-L1 levels are related to factors representing disease aggressiveness in DLBCL.

## Data Availability

The original contributions presented in the study are included in the article/supplementary material. Further inquiries can be directed to the corresponding author.
